# Involvement of Differential Relationship between HCV Replication and Hepatic PRR Signaling Gene Expression in Responsiveness to IFN-Based Therapy

**DOI:** 10.1155/2013/917261

**Published:** 2013-12-29

**Authors:** Nobukazu Yuki, Shinji Matsumoto, Michio Kato, Toshikazu Yamaguchi

**Affiliations:** ^1^Department of Gastroenterology, Osaka National Hospital, Hoenzaka 2-1-14, Chuo-ku, Osaka 540-0006, Japan; ^2^BML, Inc., Kawagoe 350-1101, Japan; ^3^Department of Gastroenterology, Minamiwakayama National Hospital, Tanabe 646-8558, Japan

## Abstract

*Aim*. To gain an insight into the effect of HCV replication-associated interference with the IFN system on hepatic mRNA expression involved in IFN production. *Methods*. Relative mRNA expression of TLR3/RIG-I signaling genes involved in IFN-**β** production was correlated with positive- and negative-strand HCV RNAs in pretreatment liver tissues responsive and nonresponsive to peginterferon and ribavirin for chronic hepatitis C genotype 1. Treatment response was analyzed for per protocol population at weeks 12 (*n* = 45) and 24 (*n* = 40) and at 24 weeks aftertreatment (*n* = 38). *Results*. HCV replication had no relation to the expression of TLR3, RIG-I, TRIF, IPS-1, IRF3, and IFN-**β** mRNAs in responders. In striking contrast, positive- and/or negative-strand HCV showed positive correlations with TLR3, RIG-I, TRIF, IPS-1, and IRF3 mRNAs in week-12 nonresponders; with RIG-I, TRIF, IPS-1, and IRF3 mRNAs in week-24 nonresponders; and with TLR3, RIG-I, and IRF3 mRNAs in posttreatment nonresponders. Thus mRNA expression of TLR3/RIG-I signaling genes was increased in relation to viral replication in nonresponders. *Conclusions*. The findings in IFN nonresponders may imply a host feedback response to severe impairment of the IFN system associated with HCV replication.

## 1. Introduction 

Upon recognition of hepatitis C virus (HCV) infection by Toll-like receptor 3 (TLR3) and retinoic-acid inducible gene I (RIG-I), the innate immune response is promptly activated in hepatocytes. The two pattern recognition receptors (PRRs) recruit their respective adaptors, Toll/interleukin-1 receptor-domain containing adaptor inducing interferon (IFN)-*β* (TRIF) and IFN-*β* promoter stimulator-1 (IPS-1), that relay the signal to downstream IFN regulatory factor-3 (IRF3), leading to the induction of IFN-*β*, known as the “front line” of host antiviral defenses in the liver [1, 2].

HCV has evolved highly successful multiple mechanisms for counteracting host antiviral responses. HCV NS3/4A serine protease in infected cells cleaves TRIF and IPS-1 and thereby disrupts the signal for IFN-*β* induction [3–5]. HCV interferes with various aspects of the downstream IFN action [6]. For example, HCV disrupts JAK-Stat signaling by NS5A and inhibits protein kinase R by NS5A and E2 proteins. Recent studies demonstrated that interference of HCV proteins with IFN production and its action depends on the levels of HCV propagation [7, 8].

Under the circumstances, we hypothesized that HCV replication-associated interference with the host IFN system may cause changes in hepatic gene expression involved in IFN production at the mRNA levels and that, if so, this host feedback response may occur in different fashion according to responsiveness to IFN-based therapy as interference with the host IFN system is considered to be more severe in nonresponders. To gain an insight into this hypothesis, we measured hepatic mRNA expression involved in IFN-*β* production, and the results were correlated with copy numbers of liver positive- and negative-strand HCV RNAs using pretreatment liver tissues responsive to IFN-based treatment and liver tissues that were nonresponsive to the treatment.

## 2. Patients and Methods 

### 2.1. Liver Tissues Responsive and Nonresponsive to IFN-Based Treatment

Liver tissues were obtained from 45 patients with chronic hepatitis C genotype 1 before 48-week treatment with weight-based doses of PEG-IFN-*α* 2b (PEG-Intron; MSD K.K., Tokyo, Japan) and ribavirin (Rebetol; MSD K.K.) [9]. A portion of the liver biopsy specimen was immediately frozen and stored at −80°C for real-time PCR. Slow virologic responders showing HCV RNA clearance after week 12 were assigned to 72-week extended treatment. Treatment response was analyzed for per protocol population at week 12 (*n* = 45), week 24 (*n* = 40), and 24 weeks aftertreatment (*n* = 38).  Table 1 summarizes the study cohort regarding achievement of complete early virologic response (cEVR) (serum HCV RNA clearance at week 12), virologic response at week 24 (VR24) (HCV RNA clearance at week 24), and sustained virologic response (SVR) (HCV RNA clearance at 24 weeks aftertreatment). The SVR group was younger and tended to be more treatment-naïve than the non-SVR group. Otherwise, no difference was seen in gender, serum alanine aminotransferase (ALT), serum HCV RNA, and liver histology. The study was approved by the local research ethics committee in accordance with the 1975 Declaration of Helsinki, and all patients provided written informed consent.

### 2.2. Hepatic mRNA Quantitation

Relative mRNA expression of TLR3, RIG-I, TRIF, IPS-1, IRF3, and IFN-*β* was determined by real-time PCR [9]. Total hepatic RNA was extracted using the TRIzol Reagent (Invitrogen, Carlsbad, CA). One *μ*g of RNA was denatured at 65°C for 5 min and reverse transcribed in a 20 *μ*L reaction mixture containing 4 *μ*L of 5× reverse-transcription (RT) buffer (Invitrogen), 0.2 *μ*mol of DTT, 100 U of Superscript II (Invitrogen), 20 U of RNasin (Promega, Madison, WI), 10 nmol of each dNTP, and 100 pmol of random hexamers. The RT reaction was performed for 10 min at 25°C, 120 min at 42°C, and then 15 min at 70°C. Primers and probes for target and reference genes studied were purchased from Applied Biosystems (Foster City, CA) (TaqMan Gene Expression Assays Hs00152933_m1 [TLR3], Hs00184937_m1 [RIG-I], Hs00706140_s1 [TRIF], Hs00325038_m1 [IPS-1], Hs00155574_m1 [IRF3], Hs0027188_s1 [IFN-*β*], and 4310884E [GAPDH]). The cDNA product was diluted 1 : 2.5, and 5 *μ*L was amplified in a 20 *μ*L reaction mixture containing 10 *μ*L of 2× TaqMan Universal PCR Master Mix and 1 *μ*L of 20× gene-specific primers and probe mixture (Applied Biosystems). PCR cycling was performed as follows: 50°C for 2 min, 95°C for 10 min, followed by 40 cycles of 95°C for 15 s and 60°C for 1 min, in an ABI PRISM 7900 Sequence Detection System (Applied Biosystems). Duplicate cycle threshold (Ct) values were analyzed by using the comparative Ct (ΔΔCt) method. The relative amount of target mRNA (2^−ΔΔCt^) was obtained by normalization to an endogenous GAPDH reference and expressed relative to the amount from normal liver tissue derived from an HCV-uninfected individual who had received hepatectomy for a metastatic liver tumor.

### 2.3. Virologic and Histologic Evaluation

HCV replication was evaluated by serum HCV RNA levels (COBAS AMPLICOR HCV MONITOR Test v.2.0, Roche Diagnostics K.K., Tokyo, Japan) and copy numbers of liver positive- and negative-strand HCV RNAs as measured by strand-specific real-time PCR [10]. Liver histology was assessed using the Knodell score [11].

### 2.4. Statistical Analysis

Data on continuous variables were presented as mean ± SD. An arbitrary value of 0 was attributed to the liver tissues negative by PCR to detect host mRNAs and viral RNAs. The range of serum HCV RNA quantitation was from 3.7 to 6.7 log IU/mL. For statistics, an arbitrary value of 7 log IU/mL was attributed to HCV RNA levels of >6.7 log IU/mL. Group comparisons were performed by nonparametric tests (Wilcoxon and Mann-Whitney) for continuous variables and by Fisher's exact test for binary variables. Spearman rank order correlations were used to study the relationship between the variables. A value of *P* < 0.05 (two-tailed) was considered to indicate significance.

## 3. Results

**Figure 1:**
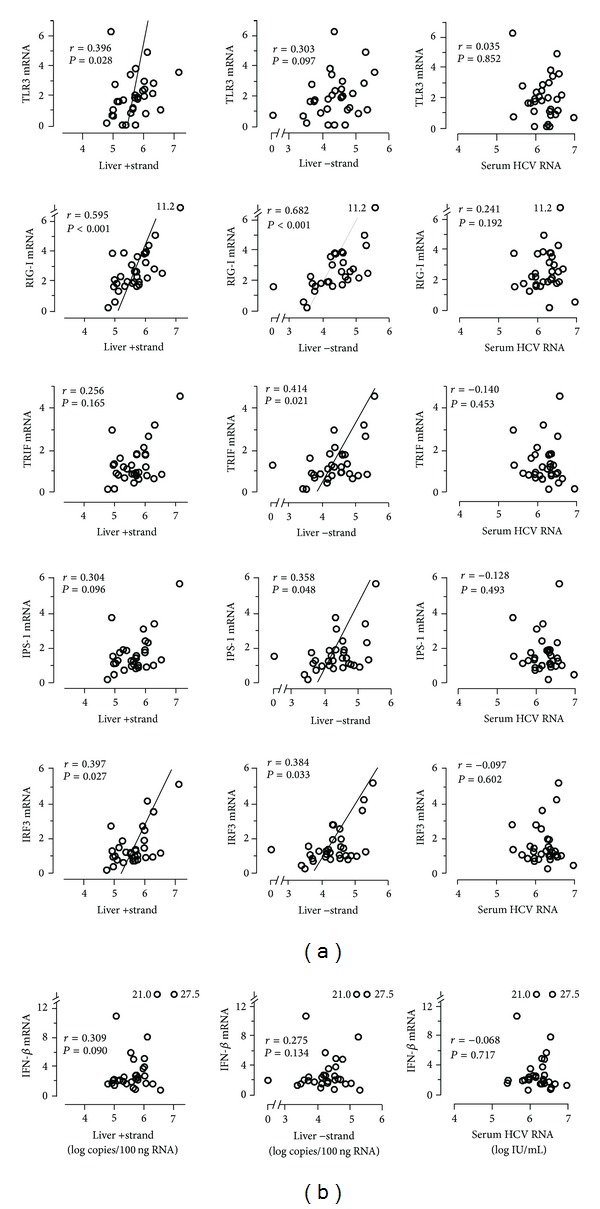
Relationship between HCV replication and hepatic PRR signaling gene expression in 31 patients nonresponsive at week 12. HCV replication was assessed by liver positive- and negative-strand HCV RNAs and circulating HCV RNA.

The relationship of hepatic PRR signaling gene expression with hepatic and circulating HCV loads was analyzed regarding responsiveness to PEG-IFN and ribavirin. In liver tissues showing cEVR, none of the mRNA expressions studied (TLR3, RIG-I, TRIF, IPS-1, IRF3, and IFN-*β*) showed a relationship with liver positive- and negative-strand HCV RNAs and circulating HCV RNA (see Supplementary Figure 1 in Supplementary Material available online at http://dx.doi.org/10.1155/2013/917261). In contrast, mRNA expression of the PRR signaling genes involved in IFN-*β* production was uniformly increased in parallel with HCV loads in liver tissues nonresponsive at week 12. Liver positive- and/or negative-strand HCV RNA(s) showed positive correlations with the mRNA levels of TLR3 (*r* = 0.396, *P* = 0.028 and *r* = 0.303, *P* = 0.097), RIG-I (*r* = 0.595, *P* < 0.001 and *r* = 0.682, *P* < 0.001), TRIF (*r* = 0.256, *P* = 0.165 and *r* = 0.414, *P* = 0.021), IPS-1 (*r* = 0.304, *P* = 0.096 and *r* = 0.358, *P* = 0.048), and IRF3 (*r* = 0.397, *P* = 0.027 and *r* = 0.384, *P* = 0.033, resp.). However, the correlations of IFN-*β* mRNA with positive- and negative-strand HCV RNAs did not reach a significant level (*r* = 0.309, *P* = 0.090 and *r* = 0.275, *P* = 0.134, resp.). Unexpectedly, these figures in nonresponders were not seen when HCV propagation was assessed by circulating HCV loads. No relationship was found between serum HCV RNA levels and any mRNA expression in liver tissues (Figure 1). Supplementary Figure  2 represents the interrelationship of liver positive- and negative-strand HCV RNAs and serum HCV RNA in the study cohort. Liver positive- and negative-strand HCV RNAs were closely correlated (*r* = 0.801, *P* < 0.001), whereas there were significant but weak correlations between serum HCV RNA and liver positive- and negative-strand HCV RNAs (*r* = 0.474, *P* = 0.001 and *r* = 0.476, *P* = 0.001, resp.).

The relationship between hepatic gene expression and HCV loads was further investigated with regard to treatment responsiveness at later time points. Like liver tissues showing cEVR, none of the mRNA expression levels showed a relationship with hepatic HCV loads when liver tissues showing VR24 and SVR were analyzed. On the other hand, the expression of a certain set of the PRR signaling genes, albeit not all, showed significant correlations with hepatic HCV loads in nonresponders. Positive- and/or negative-strand HCV RNA(s) were positively correlated with mRNA expression of RIG-I (*r* = 0.797, *P* < 0.001 and *r* = 0.744, *P* = 0.002), TRIF (*r* = 0.659, *P* = 0.010 and *r* = 0.620, *P* = 0.018), IPS-1 (*r* = 0.563, *P* = 0.036 and *r* = 0.538, *P* = 0.047), and IRF3 (*r* = 0.647, *P* = 0.012 and *r* = 0.521, *P* = 0.056, resp.) in liver tissues nonresponsive at week 24 (Table 2). When liver tissues not attaining SVR were analyzed, positive- and/or negative-strand HCV RNA(s) were positively correlated with mRNA expression of TLR3 (*r* = 0.632, *P* = 0.009 and *r* = 0.632, *P* = 0.009), RIG-I (*r* = 0.760, *P* < 0.001 and *r* = 0.693, *P* = 0.003), and IRF3 (*r* = 0.545, *P* = 0.029 and *r* = 0.426, *P* = 0.099, resp.) (Table 3). Again, the relationship of circulating HCV RNA with hepatic mRNA expression was not evident, regardless of treatment responsiveness. A significant relationship was seen only between serum HCV RNA and RIG-I mRNA expression in liver tissues without SVR (*r* = 0.511, *P* = 0.043).

## 4. Discussion

Previous studies showed that HCV proteins produced in infected cells impair the PRR signaling involved in IFN-*β* production via cleavage of TRIF and IPS-1 [3–5] and further impair downstream IFN action in various ways [6]. It has also been demonstrated that impairment of the host antiviral response by HCV depends on HCV propagation. The cleavage of IPS-1 by HCV NS3/4A serine protease is more extensive in the liver with high levels of HCV propagation [7, 8]. Hepatic mRNA expression of downstream IFN-stimulated genes (ISGs) is negatively correlated with hepatic HCV loads, indicating HCV replication-related impairment of antiviral signaling involved in ISG expression [8]. How hepatic HCV propagation is related to the expression of various PRR signaling genes involved in IFN-*β* production has not been fully clarified.

Of the PRR signaling genes, some are known ISGs (TLR3, and RIG-I), while others (TRIF, IPS-1 and IRF3) are not. We found that pretreatment hepatic mRNA expression of these genes uniformly increased in parallel with hepatic HCV loads in patients not attaining early antiviral response to PEG-IFN and ribavirin. In striking contrast, these increases were absent in responders. In IFN nonresponders, HCV replication-related increase in the mRNA expression of the PRR signaling genes was not accompanied with that in IFN-*β* mRNA expression. The mechanism underlying these findings remains unclear. Thus far, impairment of IFN production and its action has not been well studied regarding responsiveness to exogenous IFN. Our results may imply differential impairment of the host antiviral response by HCV propagation in liver tissues responsive and nonresponsive to exogenous IFN. HCV propagation may cause more severe impairment in IFN nonresponders compared with responders and, in turn, work host feedback systems to upregulate the PRR signaling gene expression involved in IFN production at the mRNA level. Further studies are needed to address these unresolved issues.

Unlike hepatic HCV loads, circulating HCV loads showed much less evident correlations with the PRR signaling gene expression in IFN nonresponders. This discrepancy may imply that circulating HCV loads do not correctly reflect HCV loads in liver tissues, which are the key compartments in which viral propagation occurs, and the HCV proteins produced interfere with IFN production and its action. Circulating HCV loads can be modified by various factors after release of HCV particles from hepatocytes, including the degree of immune clearance. Indeed, liver positive- and negative-strand HCV RNAs were closely correlated in our study cohort, whereas serum HCV RNA was weakly correlated with liver positive- and negative-strand HCV RNAs.

## 5. Conclusions

Hepatic mRNA expression of the PRR signaling genes involved in IFN production showed differential relationship with hepatic HCV loads in responders and nonresponders to IFN-based treatment. In liver tissues of nonresponders, the expression of various PRR signaling genes was uniformly increased at the mRNA levels in parallel with HCV loads. These figures were absent in liver tissues of responders. Given that interference of HCV proteins with IFN production and its action depends on the levels of HCV replication; the findings in nonresponders may reflect a host feedback response to severe HCV replication-associated impairment of the IFN system.

## Supplementary Material

The supplementary material includes supplementary Figures 1 and 2 along with their figure legends. Supplementary Figure 1 represents the data on “relationship between HCV replication and hepatic PRR signaling gene expression in patients attaining cEVR”. Supplementary Figure 2 represents the data on “interrelationship of liver positive- and negative-strand HCV RNAs and circulating HCV RNA”.

## Figures and Tables

**Table 1:** Patient characteristics regarding virologic response to PEG-IFN and ribavirin.

Characteristics	Response at week 12		Response at week 24		Posttreatment response	
	Yes	No	Yes	No	Yes	No
No.	14	31	26	14	22	16
Sex, M/F (% men)	10/4 (71)	19/12 (61)	17/9 (65)	9/5 (64)	16/6 (73)	9/7 (56)
Age	52 ± 11	59 ± 10	55 ± 11	59 ± 10	52 ± 10^a^	61 ± 8
Previous IFN therapy, *n* (%)	3 (21)	15 (48)	7 (27)	7 (50)	5 (23)^a^	9 (56)
ALT (IU/L)	85 ± 63	77 ± 41	83 ± 55	68 ± 31	89 ± 57	60 ± 29
Serum HCV RNA (log IU/mL)	6.1 ± 0.6	6.2 ± 0.4	6.2 ± 0.5	6.2 ± 0.3	6.2 ± 0.5	6.2 ± 0.3
Liver inflammatory score	7.0 ± 2.1	7.0 ± 2.5	7.0 ± 2.1	6.7 ± 2.9	7.1 ± 2.3	6.6 ± 2.7
Liver fibrosis score	1.7 ± 1.2	2.2 ± 1.1	1.9 ± 1.2	2.2 ± 1.1	1.8 ± 1.2	2.4 ± 1.0

Variables are presented as mean ± SD.

^a^Statistically significant difference *P < 0.05* between responders and nonresponders.

**Table 2:** Correlations between HCV replication (liver positive- and negative-strand HCV RNAs and circulating HCV RNA) and hepatic PRR signaling gene expression regarding virologic response at week 24.

Hepatic gene expression		Responders at week 24 (*n* = 26)			Nonresponders at week 24 (*n* = 14)		
		Liver HCV RNA		Serum HCV RNA	Liver HCV RNA		Serum HCV RNA
		+Strand	−Strand		+Strand	−Strand	
TLR3 mRNA	*r*	0.149	0.173	0.100	0.407	0.442	0.066
	*P*	0.469	0.398	0.626	0.149	0.114	0.823
RIG-I mRNA	*r*	0.217	0.324	−0.070	**0.797**	**0.744**	0.513
	*P*	0.288	0.107	0.735	**<0.001**	**0.002**	0.060
TRIF mRNA	*r*	0.014	0.091	−0.254	**0.659**	**0.620**	0.280
	*P*	0.946	0.659	0.211	**0.010**	**0.018**	0.333
IPS-1 mRNA	*r*	0.012	0.100	−0.217	**0.563**	**0.538**	0.209
	*P*	0.952	0.627	0.288	**0.036**	**0.047**	0.473
IRF3 mRNA	*r*	0.023	−0.005	−0.312	**0.647**	0.521	0.189
	*P*	0.911	0.981	0.121	**0.012**	0.056	0.517
IFN-*β* mRNA	*r*	−0.154	−0.222	−0.174	0.433	0.411	−0.022
	*P*	0.453	0.275	0.394	0.122	0.144	0.940

**Table 3:** Correlations between HCV replication (liver positive- and negative-strand HCV RNAs and circulating HCV RNA) and hepatic PRR signaling gene expression regarding posttreatment virologic response.

Hepatic gene expression		Patients with SVR (*n* = 22)			Patients without SVR (*n* = 16)		
		Liver HCV RNA		Serum HCV RNA	Liver HCV RNA		Serum HCV RNA
		+Strand	−Strand		+Strand	−Strand	
TLR3 mRNA	*r*	0.021	0.132	0.068	**0.632**	**0.632**	0.214
	*P*	0.925	0.557	0.763	**0.009**	**0.009**	0.427
RIG-I mRNA	*r*	0.192	0.243	−0.176	**0.760**	**0.693**	**0.511**
	*P*	0.392	0.277	0.432	**<0.001**	**0.003**	**0.043**
TRIF mRNA	*r*	0.109	0.200	−0.214	0.465	0.485	−0.029
	*P*	0.631	0.373	0.339	0.069	0.057	0.914
IPS-1 mRNA	*r*	0.072	0.131	−0.260	0.389	0.365	0.159
	*P*	0.750	0.562	0.243	0.137	0.165	0.556
IRF3 mRNA	*r*	0.056	0.023	−0.380	**0.545**	0.426	0.175
	*P*	0.803	0.918	0.081	**0.029**	0.099	0.516
IFN-*β* mRNA	*r*	0.034	−0.078	−0.107	0.158	0.112	−0.267
	*P*	0.882	0.730	0.634	0.560	0.680	0.318
