# Supplementary material for: Involvement of Differential Relationship between HCV Replication and Hepatic PRR Signaling Gene Expression in Responsiveness to IFN-Based Therapy
Source: Hepat Res Treat. 2013 Dec 29;2013:917261. doi: 10.1155/2013/917261 (PMC3893785; doi:10.1155/2013/917261)
Supplement: Supplementary file 1 — The supplementary material includes supplementary Figures 1 and 2 along with their figure legends. Supplementary Figure 1 represents the data on “relationship between HCV replication and hepatic PRR signaling gene expression in patients attaining cEVR”. Supplementary Figure 2 represents the data on “interrelationship of liver positive- and negative-strand HCV RNAs and circulating HCV RNA”. [file 917261.f1.pdf]

Supplement to the Manuscript

Supplementary Figure 1. Relationship between HCV replication and hepatic PRR signaling gene expression in 14 patients attaining cEVR. HCV replication was assessed by liver positive- and negative-strand HCV RNAs and circulating HCV RNA.

Supplementary Figure 2. Interrelationship of liver positive- and negative-strand HCV RNAs and circulating HCV RNA in the 45 patients studied.

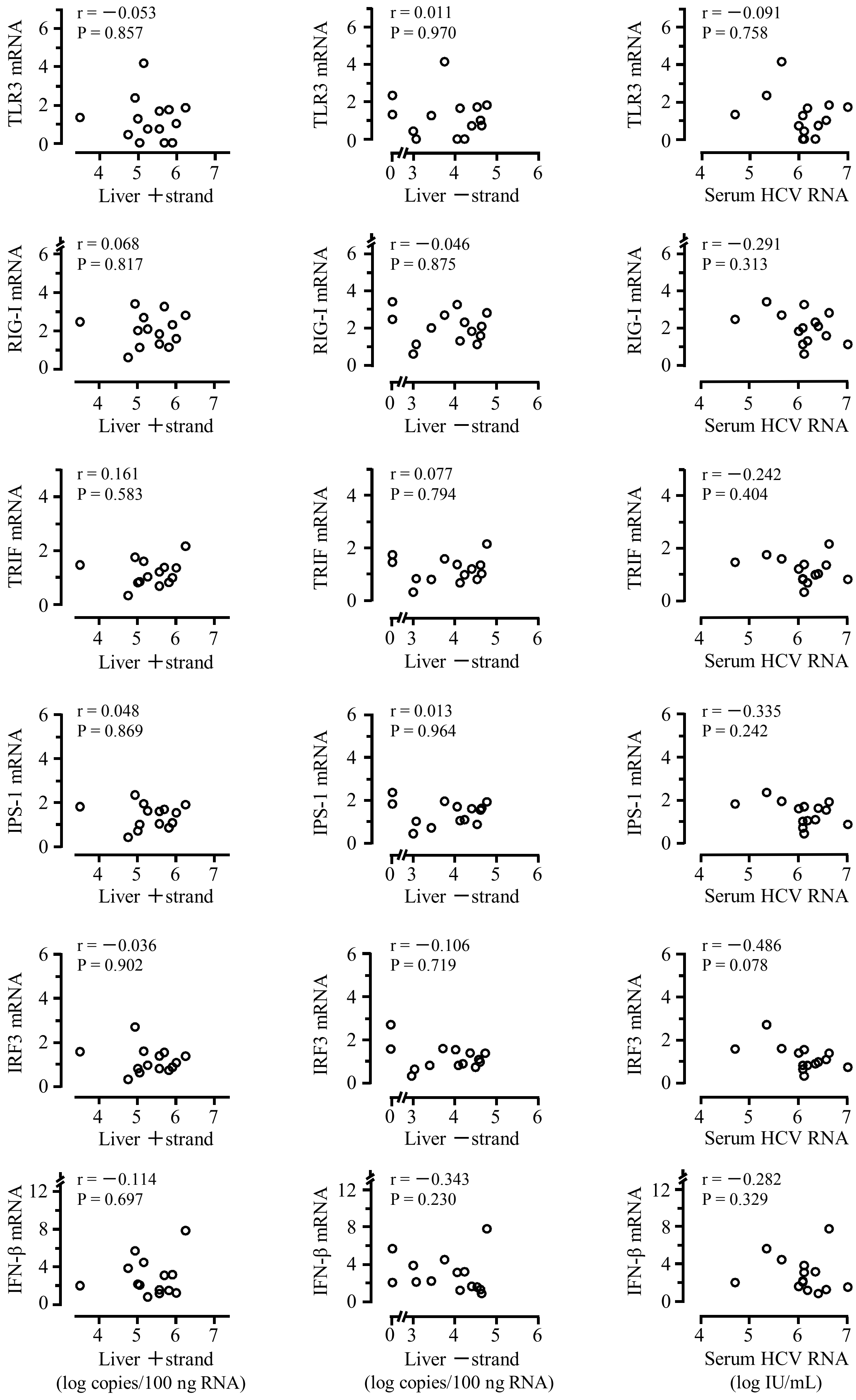

Supplementary Figure 1

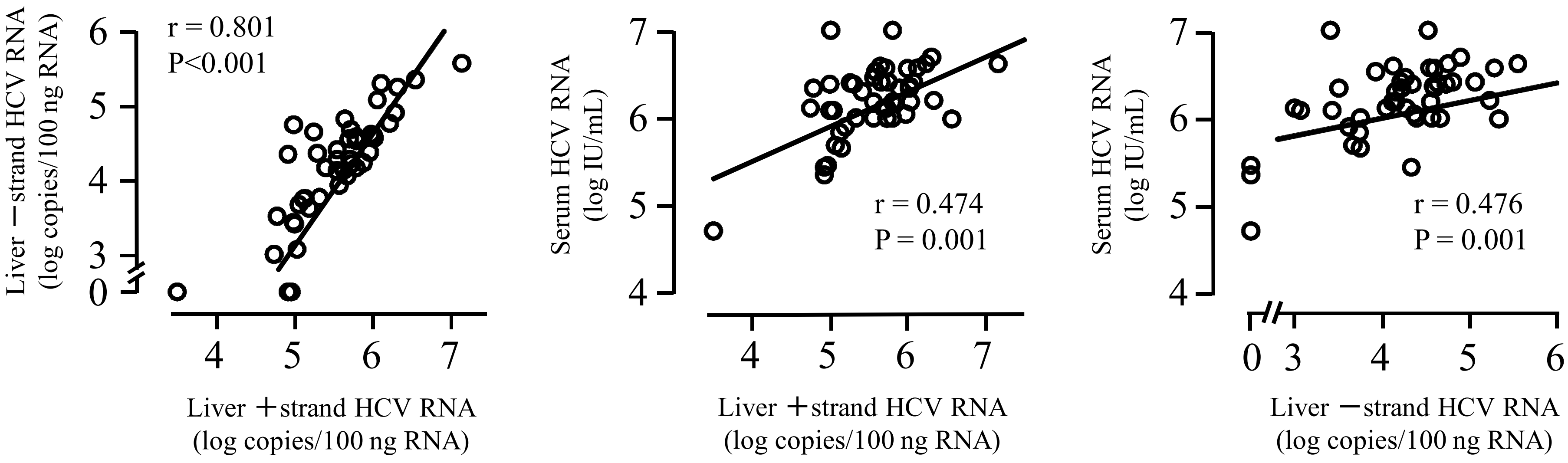

Supplementary Figure 2
